# A feasible strategy for self-assembly of gold nanoparticles *via* dithiol-PEG for photothermal therapy of cancers†

**DOI:** 10.1039/c7ra12735a

**Published:** 2018-02-07

**Authors:** Yingjie Fu, Qishuai Feng, Yajing Shen, Mengwei Chen, Chang Xu, Yu Cheng, Xiang Zhou

**Affiliations:** The Institute for Advanced Studies, Wuhan University Wuhan 430072 China xzhou@whu.edu.cn; College of Chemistry and Molecular Science, Wuhan University Wuhan 430072 China; Shanghai East Hospital, The Institute for Biomedical Engineering and Nano Science, Tongji University School of Medicine Shanghai 200029 China yucheng@tongji.edu.cn

## Abstract

We designed and explored self-assembled gold nanoparticles (SAGNPs) by introducing dithiol modified polyethylene glycol (PEG) for internanoparticle cross-linking. SAGNPs could enhance uptake into cancer cells and be disintegrated by glutathione (GSH) to achieve tumor microenvironment-activated biodegradation. This assembled structure improved the photothermal effect compared to single gold nanospheres.

## Abstract 2

We designed and explored self-assembled gold nanoparticles (SAGNPs) by introducing dithiol modified polyethylene glycol (PEG) for internanoparticle cross-linking.

## Graphical abstract

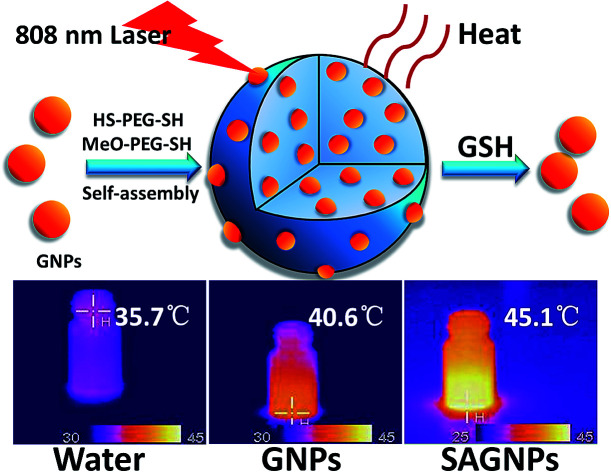

## Introduction

Gold nanoparticles (GNPs) are widely employed as drug carriers, bio-sensors and therapeutic agents.^1–3^ The chemical inertness and low toxicity of gold ensure the safety of GNPs for biological and medical applications.^4,5^ It is well known that GNPs exhibit strong interactions with light through the collective oscillations of conduction electrons resulting in surface plasmon resonance (SPR),^6^ which is tunable by size, shape and structure.^7,8^ Although spherical GNPs have already been used in the clinic, they are, unfortunately, not the optimal candidate for photothermal therapy (PTT) due to their limited absorption in the near infrared (NIR) region. One of the common methods to improve the SPR absorption is to increase the size of spherical GNPs. In addition, it was reported that large GNPs with a diameter around 70 nm exhibited enhanced cellular uptake over smaller GNPs.^9^ For *in vivo* applications, GNPs with the size under 5 nm have shown unique advantages of minimal accumulation in major organs, such as lungs, liver and spleens, as well as efficient excretion from the body through renal clearance.^10–13^ An emerging strategy to overcome the size dilemma is to assemble small GNPs into larger structures through controllable interparticle interaction to enhance the absorption of NIR light *via* distance-controlled plasmon coupling,^14–16^ while minimizing off-target organ accumulation and toxicity.

Among various documented methods to construct assembled GNPs, interparticle crosslinking is one of the most effective strategies,^17,18^ which can be readily performed by simple chemical synthesis to construct stable assembled GNPs. These linkers efficiently shorten the distance between GNPs and keep the assemblies stable in aqueous solutions. For biomedical applications, it requires the linkers are either biodegradable or collapsed selectively to the tumor microenvironment *in vivo*^19–21^. Therefore, a “smart” linker to precisely control assemble and disassemble of GNPs is critical for efficient *in vivo* delivery and therapy.

**Scheme 1:**
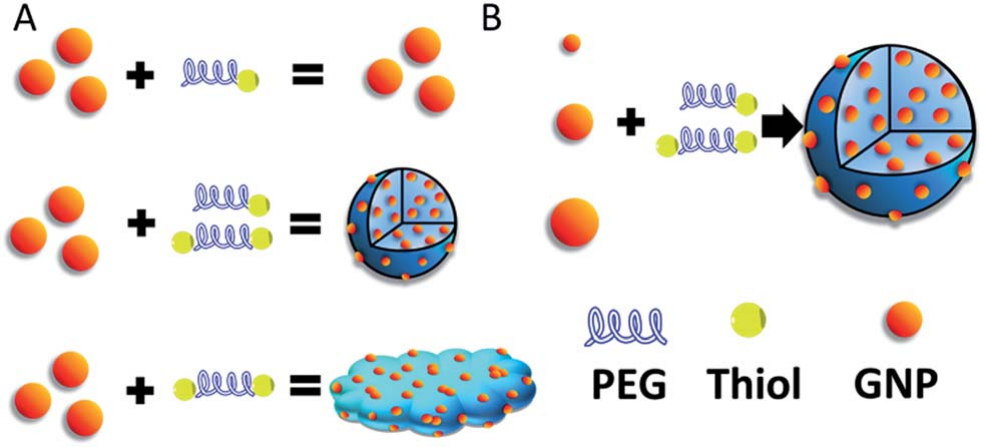
(A) Construction of SAGNPs. (B) GNPs can form similar assembled structures which is independent of the particle size.

In our previous work, the assembled GNPs were utilized as a nanocarrier for brain tumor treatment.^22^ However, the formation mechanism and internal structure of SAGNPs are not clear yet. Whether this strategy could be utilized commonly or specifically for constructing assemblies is also unknown. Thus, in this work, the dithiol functionalized PEG was used as a cross-linker to assemble GNPs through S–S bonds which are stable under normal physiological conditions, but can be selectively cleaved by GSH which is highly enriched in cancer cells.^23,24^ We further explored the formation mechanism by adjusting the ratio of reactant and confirmed the linkage between particles, and applied this method to other gold nano-assemblies construction (Scheme 1). Additionally, in contrast to the GNPs, the increased size of assembled SAGNPs are promising to enhance the uptake of nanoparticles by cancer cells, as well as the absorption at NIR to improve the therapeutic efficacy of PTT.

## Results and discussion

**Fig. 1:**
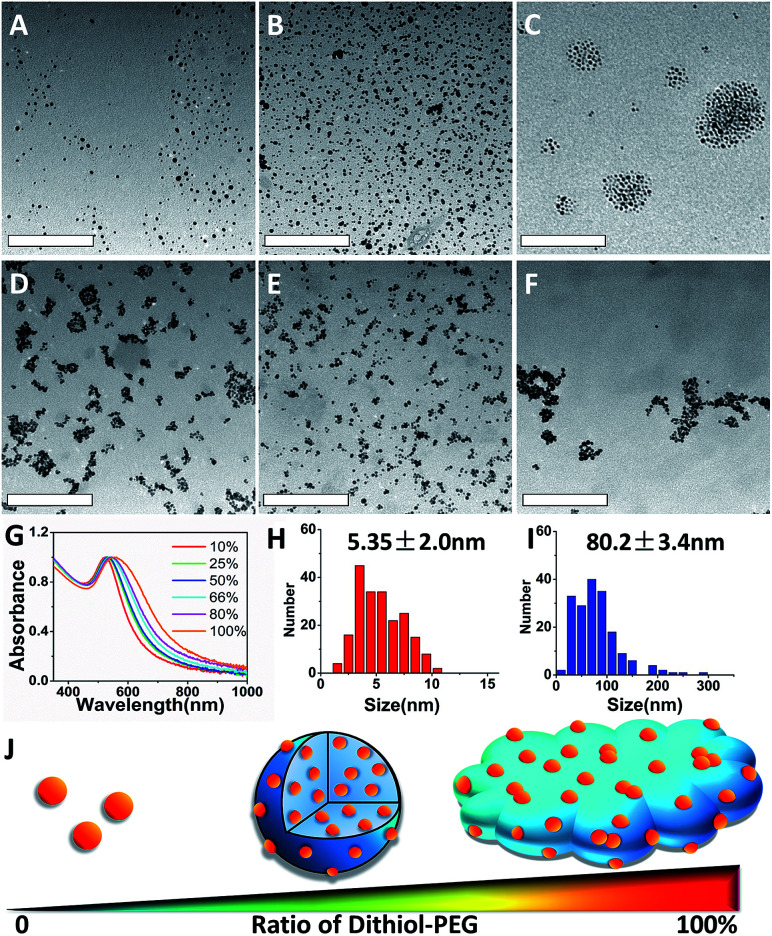
TEM images of GNPs with different percentage of dithiol-PEG: (A) 10%, (B) 25%, (C) 50%, (D) 66%, (E) 80% and (F) 100%. The scale bar is 200 nm. (G) UV-vis spectra of GNPs with various dithiol-PEG ratios. Average diameters of (H) dispersed GNPs and (I) SAGNPs based on TEM. (J) Scheme of various structures according to the ratio of dithiol-PEG.

The SAGNPs were readily synthesized through interparticle crosslinking using dithiol-modified PEG as the linker. To understand the formation of SAGNPs, we explored the influence of different ratios of thiol-PEG_5000_-thiol (dithiol-PEG) and MeO-PEG_5000_-thiol (thiol-PEG) reacting with GNPs, while the molar ratio of total PEG chains to GNPs was kept at 500 : 1. As shown in Fig. 1, different morphologies of assembled nanoparticles were observed based on initial ratios of dithiol-PEG/thiol-PEG. There was no SAGNP formed when the percentage of dithiol-PEG was below 50% (Fig. 1A–C). The diameter of assembled SAGNPs increased to 80.2 ± 3.4 nm, compared to the 5.4 ± 2.0 nm average size of single GNPs, as measure by TEM (Fig. 1H and I). Interestingly, as the ratio of dithiol-PEG increased, the size of crosslinked SAGNPs was significantly decreased with some non-spherical aggregates appeared (Fig. 1D). When the proportion of dithiol-PEG was over 80%, very low amount of spherical assemblies could be observed (Fig. 1E and F). The high density of thiol groups led to large polymeric complex and extra thiol groups on GNPs induced aggregation. Moreover, an obvious enhanced absorption at NIR region was observed and correlated with the increased ratio of dithiol-PEG, as shown by the UV-vis spectroscopy (Fig. 1G). It has been proved that the SPR band can be changed in assembled GNPs by the close interaction of adjacent GNPs and enhance photothermal effect.^25–28^

**Fig. 2:**
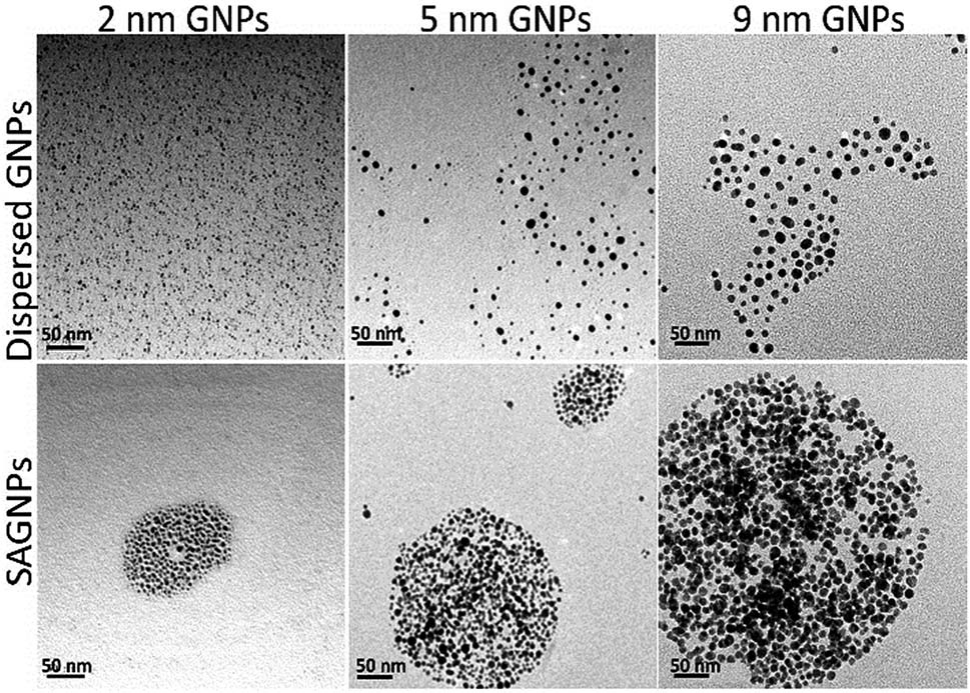
GNPs with different sizes formed similar assemblies. Dispersed GNPs were reacted with thiol-PEG; SAGNPs were reacted with thiol-PEG and dithiol-PEG (ratio: 1 : 1).

To test the capability of this assembly strategy, GNPs with 2 nm and 9 nm were also used to build the nano-assemblies. Indeed, there GNPs can form gold nano-assemblies with spherical morphology when the percentage of dithiol-PEG was 50% (Fig. 2). The assemblies all had the red-shift of SPR band and enhanced absorption in the NIR region (Fig. S1†). GNPs before assembling were mono-dispersed proved by DLS analysis and showed no obvious influence on UV-vis spectra (Fig. S2†). To investigate how the dithiol/thiol-PEG controlled the morphology of gold nano-assemblies, varying ratios of two types of PEGs were mixed without gold nanoparticles. TEM images illustrated that low percentage of dithiol-PEG (<50%) only formed small debris, while when the dithiol-PEG reached 50%, spheres were formed, and larger polymer complexes were observed when the ratio of dithiol-PEG continued increasing (Fig. S3†), which is consistent with the morphology change of gold assemblies. To further prove the formation of S–S bond is a critical mechanism of the self-assembly of SAGNPs, we kept PEG away from oxygen, deferring the oxidation of thiols to form S–S linkers, since oxidant is known necessary to form the S–S bond.^29,30^ No spherical nanoparticles were formed by PEGs using any proportion of PEGs (Fig. S3†). However, when excess oxides were introduced by hydrogen peroxide, crosslinking was dramatically increased along with the increasing percentage of dithiol-PEG (Fig. S3†). As a result, spherical gold assemblies were supported by oxidized S–S linkers and the reaction conditions were optimized to construct SAGNPs.

**Fig. 3:**
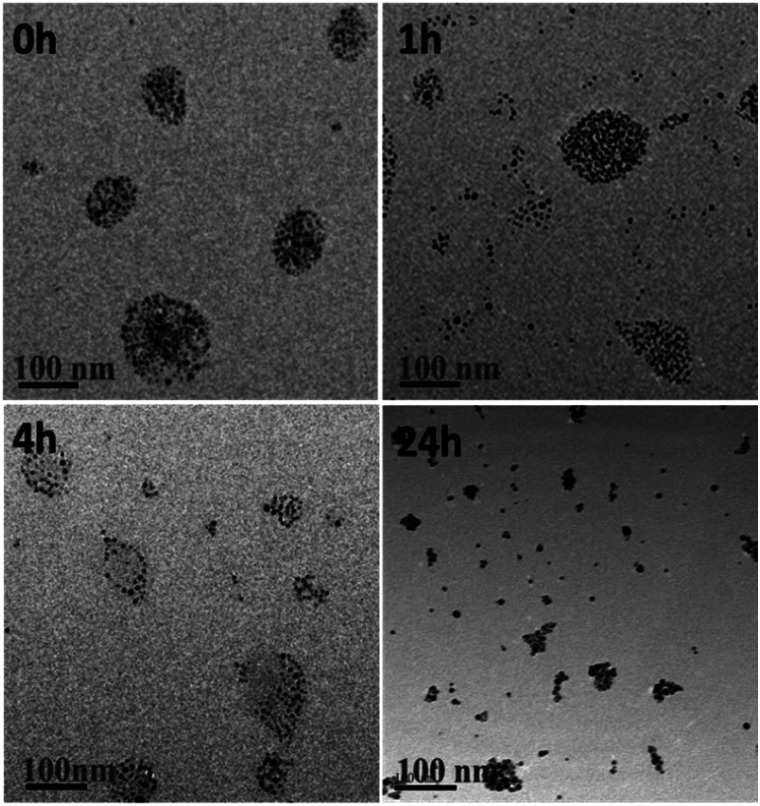
TEM images of GSH induced collapse of SAGNPs. The incubation time was 0 h, 1 h, 4 h and 24 h. The scale bar is 100 nm.

To illustrate intracellular GSH could collapse the assemblies, SAGNPs were incubated with 10 mM of GSH at 37 °C. As expected, SAGNPs were gradually disassembled into small pieces and finally converted to single GNPs within 24 h (Fig. 3), but no obvious disassociation of SAGNPs was observed at GSH concentrations lower than 1 mM (Fig. S4†). By contrast, thiol-PEG modified GNPs (mPEG-GNPs) were dispersed well in PBS, and formed small aggregates after incubation with GSH (Fig. S5†). These data confirmed that SAGNPs remained stable and intact under physiological conditions, but respond well to high GSH concentrations to fall apart into small gold particles, which may contribute to a satisfied safety profile.^31,32^

**Fig. 4:**
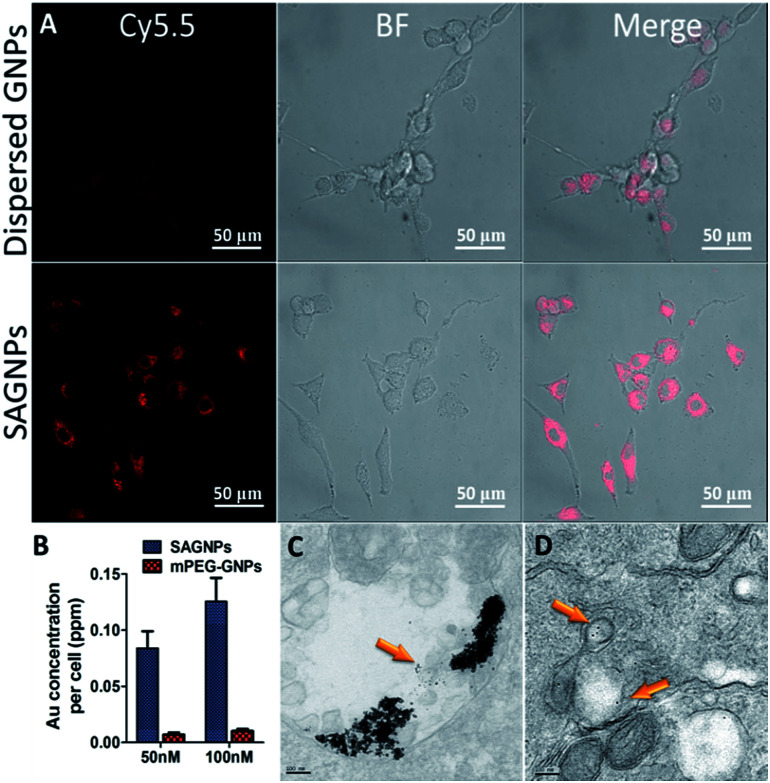
(A) Fluorescence images of U87 cells after incubation with Cy5.5 labelled GNPs and SAGNPs for 24 h. (B) ICP-MS analysis of Au cellular uptake incubated with 50 nM and 100 nM of SAGNPs and mPEG-GNPs for 24 h. Cell TEM of (C) SAGNPs, (D) mPEG-GNPs, cells were incubated with 50 nM SAGNPs and GNPs for 24 h.

As a drug delivery platform, efficient cell uptake is important to ensure sufficient therapeutic outcomes.^33^ SAGNPs were labelled with Cy5.5 (Fig. S6†), and incubated with U87 human glioma cells for 24 h. As shown by fluorescence imaging, SAGNPs showed dramatically enhanced internalization into cancer cells over single GNPs (Fig. 4A). The Au content in the cells was further quantified by ICP-MS (Fig. 4B). Cells endocytosed 12 folds more gold than dispersed GNPs after incubated with 50 nM SAGNPs for 24 h. Gold content in the cells increased with the concentration of SAGNPs, consistently, 12.3 folds more gold were observed after doubled the particles concentration (Fig. 4B). It has been reported that assembled nanoparticles with larger particle size could be stunk inside tumor cells with higher accumulation and lower exocytosis to improve therapeutic outcome.^17^ It is consistent with our cell TEM images which illustrated enhanced accumulation of SAGNPs in cytoplasm than mPEG-GNPs (Fig. 4C and D). Additionally, cell TEM imaging also confirmed the accumulation of SAGNPs inside the cell with some single GNPs collapsed from SAGNPs after 24 h incubation (Fig. 4C).

**Fig. 5:**
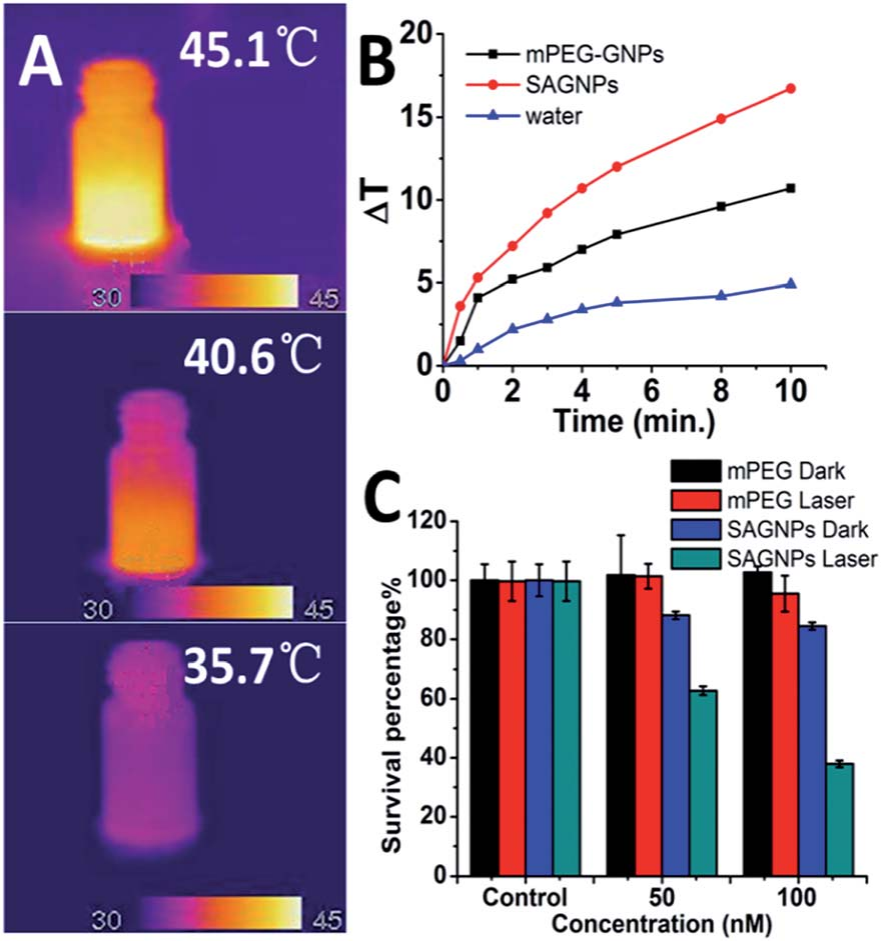
(A) Thermal images and (B) temperature raise curve of SAGNPs, mPEG-GNPs and water treated with 2 W cm^−2^ 808 nm laser. (C) PTT results of SAGNPs *via* U87 cells.

One of the major advantages of SAGNPs is the enhancement in absorption at NIR to improve the therapeutic efficacy of PTT. After irradiation by laser at 808 nm, SAGNPs exhibited 16.7 °C temperature raise comparing to dispersed GNPs (10.7 °C) and water (4.9 °C) (Fig. 5A and B). The same phenomena were observed when irradiating SAGNPs of different particle sizes (Fig. S7†). To investigate the PTT efficiency of SAGNPs, U87 cells were incubated with different NPs for 24 h and received irradiation at 808 nm. According to the previous data, SAGNPs were well enriched in cancer cells (Fig. 4B) and were not totally collapsed by GSH after 24 h incubation (Fig. 4C). The cells were efficiently damaged *via* hyperthermia caused by SAGNPs after 20 min 2 W cm^−2^ irradiation (Fig. 5C, S8†). As a control, mPEG-GNPs did not cause obvious toxicity (Fig. 5C, S8†). Taken together, after assembly, SAGNPs significantly improved the photothermal conversion efficiency of GNPs, which leads to enhanced therapeutic effect of PTT.

## Experimental

### Synthesis of GNPs

5 nm GNPs were synthesised according to our previous work. Briefly, 0.25 mM of tetra-*n*-octylammonium bromide (TOAB) and 0.6 mM of dodecylamine (DDA) were dissolved together in 5 mL of toluene. 366 μL of 0.57 M HAuCl_4_ (17% in HCl solution) was added and vigorously stirred to obtain a stable solution. Then, 75.66 mg of NaBH_4_ was dissolved in 1 mL of ice-cold pure water and added slowly over 2 min with vigorous stirring. After 2 h reaction, the mixture was poured into 40 mL of ethanol. The precipitate was collected by centrifugation at 4000 rpm for 10 min for two times and redispersed in 4 mL of chloroform.

#### 2 nm GNPs

1.0 g of HAuCl_4_ and 150 mL of pure water was mixed and stirred slowly for 5 minutes to obtain a clear yellow solution. Premix 2.1 g of tetra-*n*-octylammonium bromide in 150 mL of toluene and add it to the gold solution. Then, the mixture was stirred vigorously for 15 minutes and added 0.7 mL of 1-pentanethiol dropwise over 15 minutes. Stirred until the solution presents a cloudy deep white and quickly added a freshly prepared solution of 2.0 g of sodium borohydride in 10 mL pure water. The reaction lasted overnight and removed the water phase. Evaporated most of the solvent and washed with ethyl alcohol for 5 times.

#### 9 nm GNPs

100 mL 0.3 mM of HAuCl_4_ solution were added 1 mL 0.4 M of sodium citrate and stirred rapidly for 5 min. Then, 0.5 mL 0.132 M of fresh made NaBH_4_ was poured into the solution quickly and kept stirring for 1 h.

### Synthesis of PEGylated GNPs

GNPs were mixed with PEG in 3 mL chloroform and stirred for 48 hours uncapped. The different ratios of two PEGs were listed in Table S1.† Then, PEGylated GNPs were dried under vacuum and redispersed in water. GNPs were collected *via* centrifugation at 4500 rpm in centrifuge tubes (filter membrane cutoff 10 kDa) and washed with ultra-pure water for three times.

### PEG reactions

Two kinds of PEGs were mixed according to the ratios as Table S1† showed. 3 mL of chloroform were added and stirred without capping for 48 h. Oxygen-free condition was realized by protection of Ar atmosphere and PEGs reacted in sealed bottles. Oxygen-rich condition was realized by adding 10 μL of 30% hydrogen peroxide in the reaction for 48 h.

### GSH induced disassembling

SAGNPs were diluted to 50 nM and incubated with GSH with concentration of 10 mM, 1 mM, 0.1 mM, 0.01 mM and 0.001 mM.

### ICP-MS analysis for cellular uptake

U87 cells were seeded in 6-wells plates, and incubated with 50 nM of SAGNPs and mPEG-GNPs separately. Cells were calculated by flow cytometry analysis. Then, purified cells were digested by aqua regia.

### Confocal microscopy

U87 cells were plated in a culture dish at a concentration of 5 × 10^4^ cells per dish and incubated for 24 h before treatment. The cells were then incubated with Cy5.5 labelled SAGNPs for 24 h at 37 °C. The cells were stained with 75 nM LysoTracker green for 30 min. The cells were washed with PBS twice, and the fluorescence distribution was visualized *via* confocal microscopy.

### Cell TEM

Cells were cultured and incubated with GNPs and SAGNPs for 24 h separately. Then the cells were fixed and harvested by centrifugation. After incubated with 1% osmium tetroxide for 60 min, the cells were stained in 1% uranyl acetate in maleate buffer. After dehydration and infiltration, the embedded cells were polymerized and spurred in a 60 °C oven for 2 days. Then, 90 nm sections were cut using a Reichert–Jung Ultracut E and stained with uranyl acetate and citrate. The images were captured at 300 kV using a FEI Tecnai F30, Gatan CCD digital micrograph.

### Photothermal effect and photothermal therapy

GNPs and SAGNPs were diluted to 50 nM. Laser at 808 nm was irradiated for 10 min with power of 2 W cm^−2^. U87 glioma cells were seeded in 96-well plate and incubated with 50 nM of GNPs and SAGNPs separately. After 24 h incubation, the cells receipt 2 W cm^−2^ irradiation of 808 nm and incubated for another 24 h. Then, cells were calculated by CCK-8 analysis.

## Conclusions

In summary, we designed and explored a feasible self-assembled drug delivery platform, SAGNPs, using dithiol-PEG as the cross-linker. The assembled structure remained stable under physiological conditions, improved cellular uptake and cell retention, and, more importantly, enhanced photothermal effect by enhancing NIR absorbance. Due to the sensitivity of S–S bond to GSH, SAGNPs responded to the acidic environment to collapse back into small GNPs for satisfied biocompatibility and safety. Moreover, free thiol groups on SAGNPs are readily to conjugation with probes, targeted molecules, and therapeutic agents for theranostic aims. This work provides a simple method of assembly which is appropriate to GNPs with various sizes. This new nanodelivery platform may hold a promise for precise diagnosis and efficient therapy in clinic practice.

## Conflicts of interest

There are no conflicts to declare.

## Supplementary Material

RA-008-C7RA12735A-s001
