# Supplementary material for: A feasible strategy for self-assembly of gold nanoparticles via dithiol-PEG for photothermal therapy of cancers
Source: RSC Adv. 2018 Feb 7;8(11):6120–4. doi: 10.1039/c7ra12735a (PMC9078271; doi:10.1039/c7ra12735a)
Supplement: RA-008-C7RA12735A-s001 [file RA-008-C7RA12735A-s001.pdf]

Electronic Supplementary Material (ESI) for RSC Advances.
This journal is © The Royal Society of Chemistry 2018

Electronic Supplementary Information (ESI)

A Feasible Strategy for Self-Assembly of Gold Nanoparticles via Dithiol-PEG for Photothermal Therapy of Cancers

Yingjie Fu[a,b], Qishuai Feng[c], Yajing Shen[c], Mengwei Chen[c], Chang Xu[c], Yu Cheng[c]* and Xiang Zhou[a,b]*

a. The Institute for Advanced Studies, Wuhan University, Wuhan 430072, China

b. College of Chemistry and Molecular Science, Wuhan University, Wuhan 430072, China

c. Shanghai East Hospital, The Institute for Biomedical Engineering and Nano Science, Tongji University School of Medicine, Shanghai 200029, China

Contents

General information • 2

Supporting experiments and figures • 2-5

General information

All materials were obtained from commercial suppliers without further purification. Ultrapure water was used in all experiments. UV-vis spectra were recorded by Cary 60 UV-Vis, Agilent Tech. TEM photos were obtained from Tongji University (JEOL 1230, Japan). Colocalization was measured by confocal microscopy (LeicaTCS SP5). U87 cells were obtained from Shanghai East Hospital.

Supporting experiments and figures

Thiol-PEG_{5000}-thiol percentage	GNPs	Thiol-PEG_{5000}-thiol	MeO-PEG_{5000}-thiol
0	2×10^{-8} mol	0	50mg, 10^{-6} mol
10%	2×10^{-8} mol	5mg, 10^{-7} mol	45mg, 9×10^{-7} mol
25%	2×10^{-8} mol	12.5mg, 2.5×10^{-7} mol	37.5mg, 7.5×10^{-7} mol
50%	2×10^{-8} mol	25mg, 5×10^{-7} mol	25mg, 5×10^{-7} mol
66%	2×10^{-8} mol	33mg, 6.6×10^{-7} mol	17mg 3.4×10^{-7} mol
80%	2×10^{-8} mol	40mg, 8×10^{-7} mol	10mg, 2×10^{-7} mol
100%	2×10^{-8} mol	50mg, 10^{-6} mol	0

Table S1. Synthetic method of PEGylated GNPs with different composite ratio. GNPs were quantified via UV-Vis spectrum.

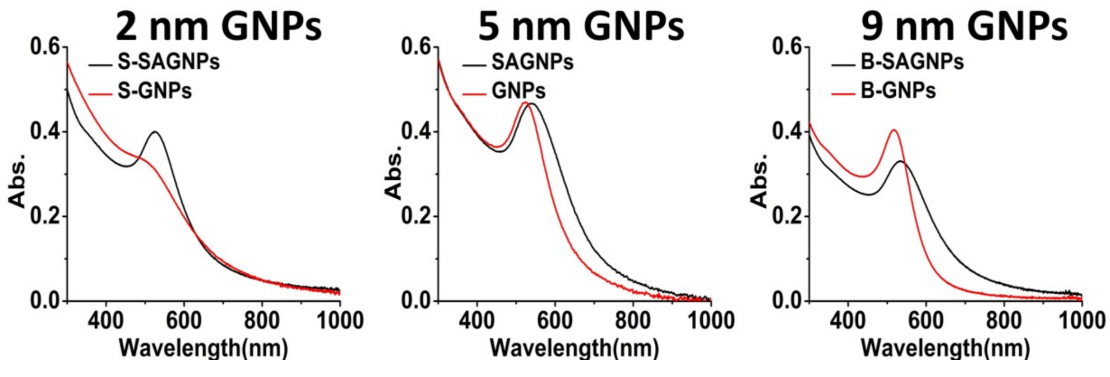

Figure S1.UV-Vis spectra of GNPs and SAGNPs of different sizes.

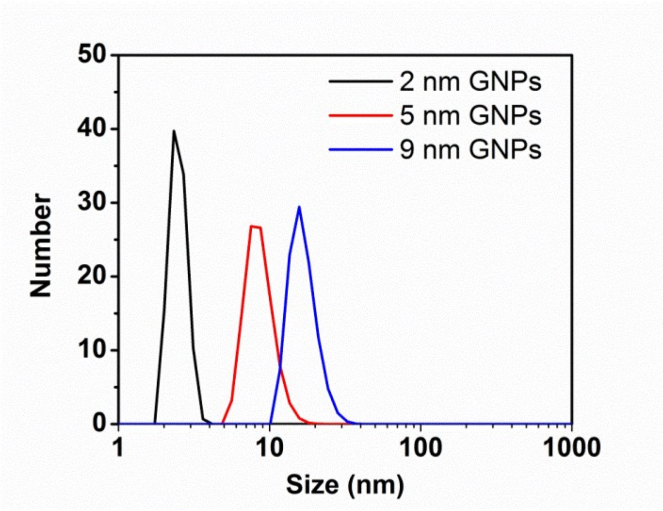

Figure S2. Size distribution analysis based on DLS.

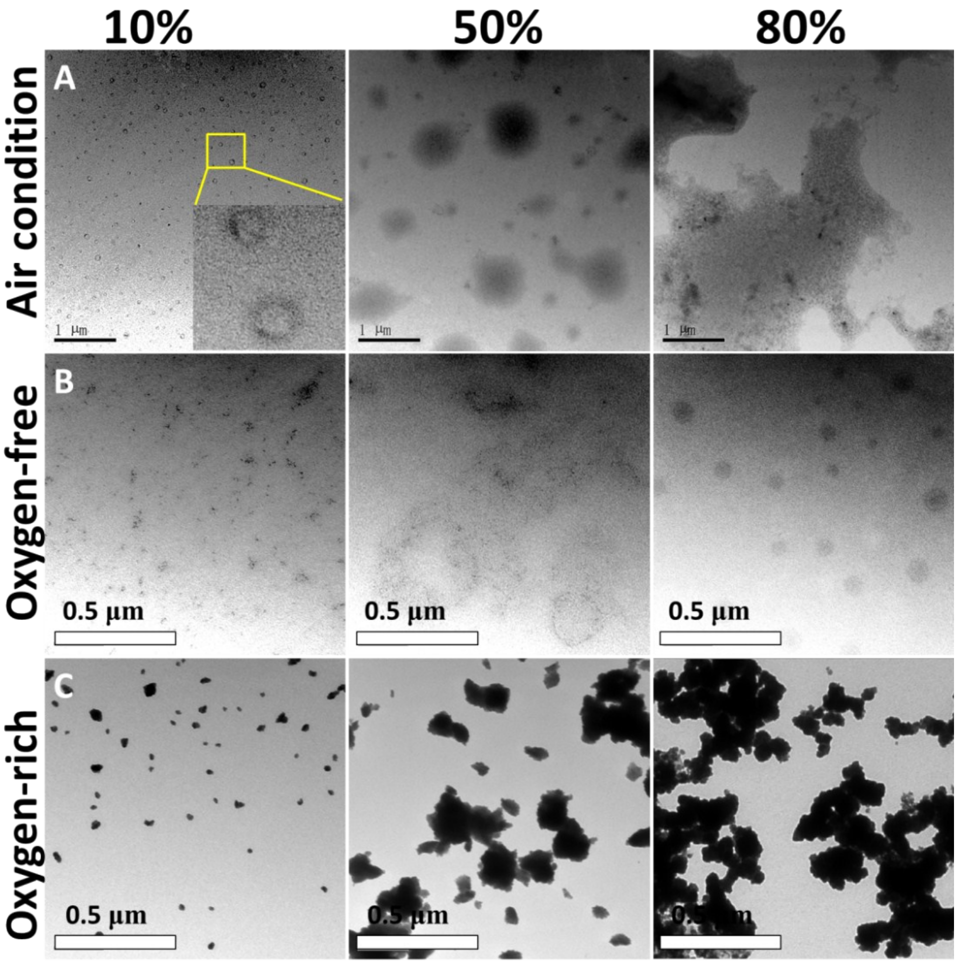

Figure S3. TEM images of PEG mixture after 24 h reaction and negative staining. Percentage of dithiol-PEG: 10%, 50%, and 80%. (A) PEGs of different ratios mixed open to the air without GNPs. The scale bar is 1μm. (B) Oxygen-free reaction of PEG with different ratios after 48 h. (C) Oxygen-rich reaction of PEG with 0.1 mM of hydrogen peroxide. The scale bar is 0.5 μm.

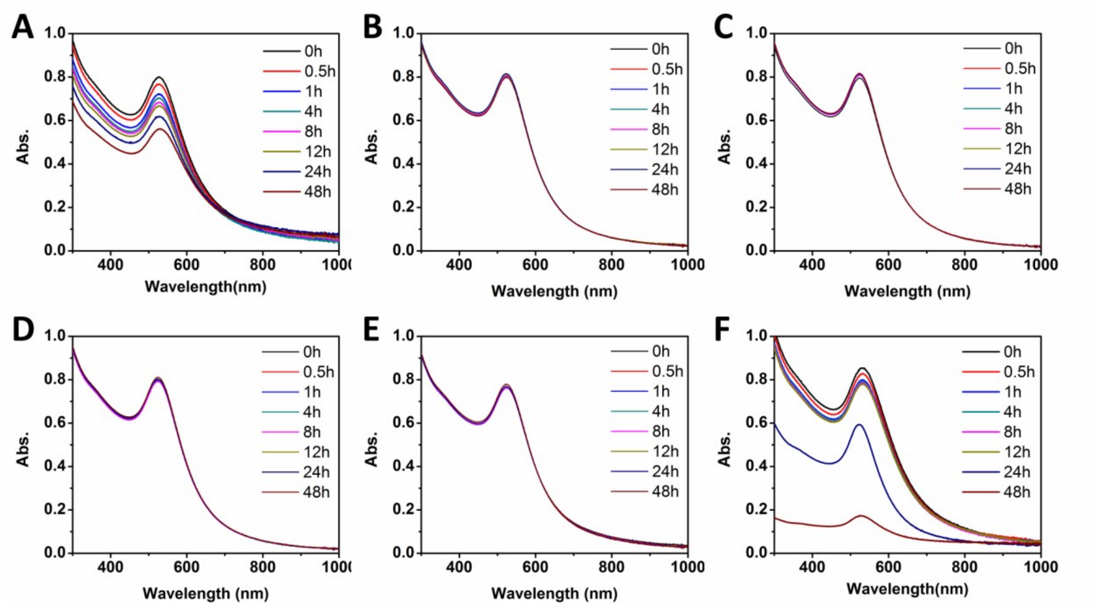

Figure S4. UV-Vis spectra of SAGNPs incubated with GSH at (A) 10 mM, (B) 1 mM, (C) 0.1 mM, (D) 0.01 mM, and (E) 0.001 mM. (F) UV-Vis spectra of SAGNPs reacted with Tris(2-carboxyethyl)phosphine (TCEP).

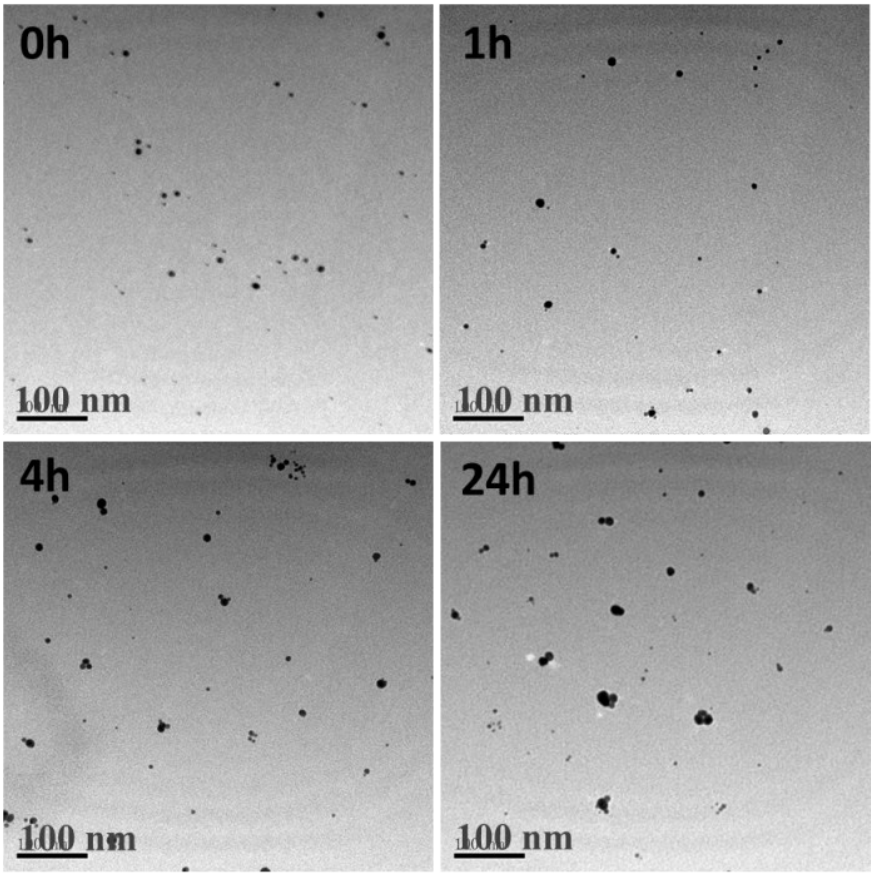

Figure S5. TEM images of mPEGs-GNPs reacted with 10 mM GSH.

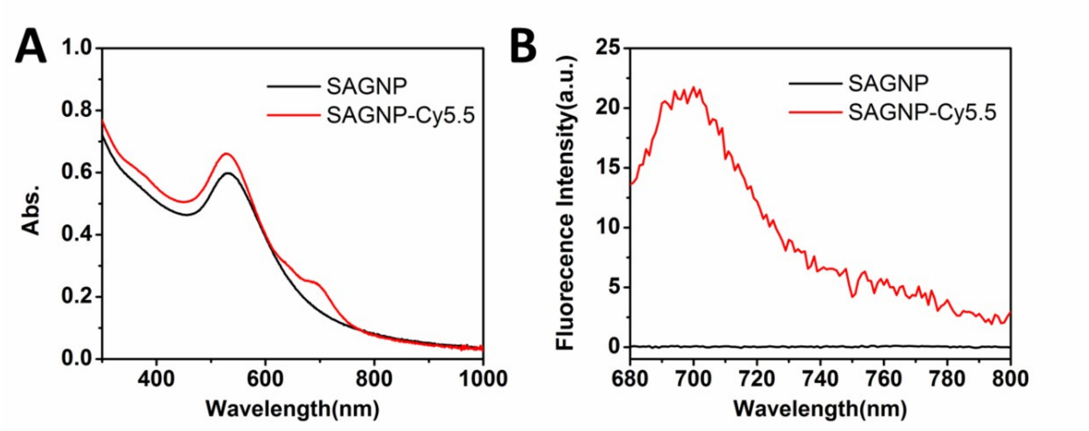

Figure S6. The comparison of (A) UV-Vis spectra and (B) fluorescence spectra between SAGNPs and SAGNP-Cy5.5

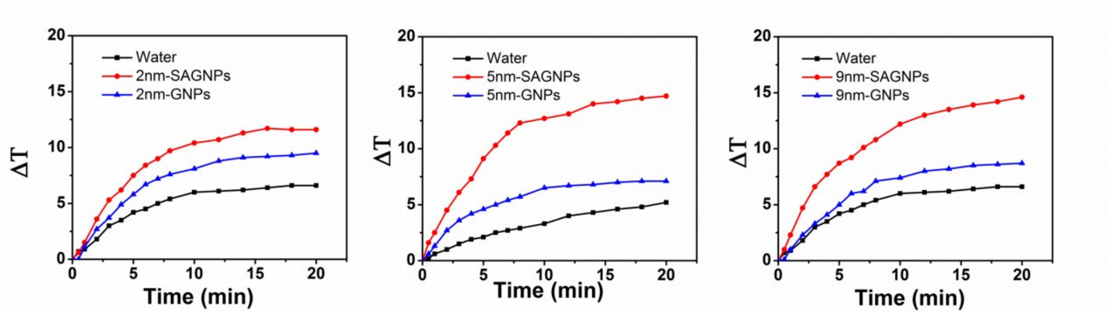

Figure S7. (A) Cytotoxicity based on MTT assays of mPEG-GNPs and PEG. The amount of PEG was related to the molar ratio of SAGNPs by 500:1. (B) Cytotoxicity of SAGNPs on different cell lines.

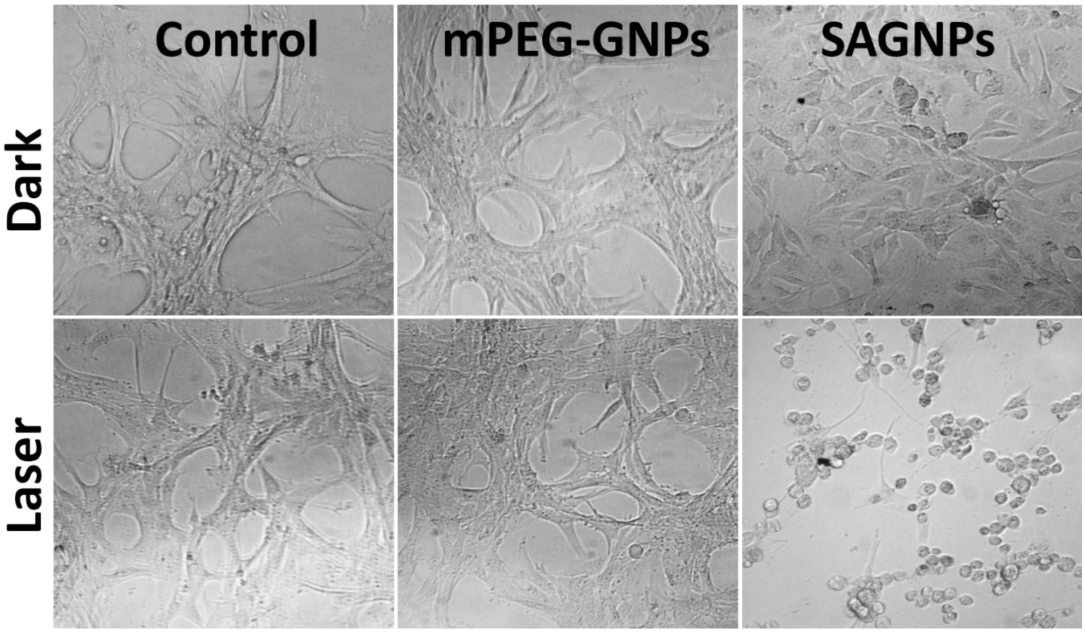

Figure S8. Changes of cell state after irradiation.
